# Efficacy of vitamin D supplementation in patients diagnosed with depression: a dose–response meta-analysis of randomized controlled trials

**DOI:** 10.3389/fnut.2026.1772451

**Published:** 2026-03-17

**Authors:** Hsuan-Hsien Liu, Ting-Hui Liu, Chia-Yu Liu, Jheng-Yan Wu, Chien-Ho Lin, Chih-Cheng Lai

**Affiliations:** 1 Department of General Medicine, Linkou Chang Gung Memorial Hospital, Taoyuan, Taiwan; 2 Department of Psychiatry, Chi Mei Medical Center, Tainan, Taiwan; 3 Department of Audiology and Speech-Language Pathology, Asia University, Taichung, Taiwan; 4 Department of Nutrition, Chi Mei Medical Center, Tainan, Taiwan; 5 Department of Public Health, College of Medicine, National Cheng Kung University, Tainan, Taiwan; 6 Department of Intensive Care Medicine, Chi Mei Medical Center, Tainan, Taiwan; 7 School of Medicine, College of Medicine, National Sun Yat-sen University, Kaohsiung, Taiwan

**Keywords:** depression, dose–response, meta-analyze, randomized clinical trial, vitamin D

## Abstract

**Background:**

Depression affects 5% of the global population, posing significant health and economic challenges.

**Objectives:**

This study evaluates the efficacy of vitamin D supplementation in reducing depressive symptoms and explores its dose–response relationship.

**Methods:**

We used PubMed, EMBASE, and Cochrane Library to identify randomized controlled trials using the keyword combination of vitamin D and depression from inception to June 2024. The primary outcome was the change in depressive symptoms. A dose–response meta-analysis using restricted cubic splines was conducted to explore potential sources of heterogeneity and examine the dose–response relationship.

**Results:**

The outcomes were reported in 15 studies encompassing data from 962 participants. Vitamin D supplementation demonstrated a significant improvement in depressive symptoms compared to the placebo group (SMD: −0.98; 95% CI − 1.28 to −0.68; *p* < 0.001). Statistical heterogeneity was high (*I^2^* = 79%; *p* < 0.001). Secondary outcomes revealed significant reductions in serum PTH (MD: −4.19; 95% CI − 8.18 to −0.2 pg./mL) and TNFα levels (MD: −0.3; 95% CI − 0.44 to −0.16 pg./mL) in the intervention groups, while other outcomes, such as BMI, weight, and IL-6, showed no significant changes. Dose–response analysis further highlighted that higher daily doses of vitamin D, particularly up to 5,000 IU/day, were associated with the greatest reduction in depressive symptoms.

**Conclusion:**

Our findings from this systematic review and meta-analysis indicated that vitamin D supplementation may be an effective adjunctive therapy for improving depressive symptoms. The observed reductions in serum PTH and TNFα levels suggest anti-inflammatory mechanisms underlying its antidepressant effects. Higher daily doses, particularly around 5,000 IU, were associated with greater symptom improvement within the studied populations.

## Introduction

Depression is a prevalent and severe mental disorder that poses significant health challenges worldwide. According to the World Health Organization, approximately 5% of the global population suffers from depression, contributing to widespread impacts on individuals’ quality of life and considerable social and economic burdens (1). Depression manifests through various clinical symptoms, including persistent low mood, anhedonia, sleep disturbances, diminished self-esteem, somatic complaints, preoccupation with death, and suicidal ideation (2). Current mainstream interventions for depression include pharmacotherapy and psychotherapy. However, a substantial proportion of patients respond inadequately to these conventional treatments (3). Moreover, a high recurrence rate of the disorder further complicates long-term outcomes, even among those who achieve remission. The widespread prevalence of depression combined with its low remission rate highlights the condition’s significance as a major public health concern.

Vitamin D is a lipid-soluble vitamin with multiple functions crucial for human health. It is well-known for its ability to promote the absorption of calcium and phosphorus, which are essential for maintaining healthy bones and teeth (4). The role of vitamin D in the brain has gradually attracted the attention of the scientific community. Research indicates that vitamin D is involved in the development and regulation of the nervous system (5). Vitamin D receptors are widely distributed in the brain, particularly in areas that regulate behavior and emotions, such as the hippocampus and hypothalamus (6). The presence of these receptors suggests that vitamin D may have important functions in the brain. Furthermore, vitamin D has anti-inflammatory and anti-oxidant properties (7) that are crucial for protecting nerve cells from damage and reducing neuroinflammation. Current research has indicated a correlation between vitamin D deficiency and depression (8, 9). Epidemiological studies have also demonstrated an association between vitamin D deficiency and depression (10, 11). This association raises the question about whether vitamin D supplementation can improve depressive symptoms. Compared with antidepressant medications, vitamin D supplementation is generally considered well tolerated and is associated with a favorable safety profile (12). However, current research results on this topic remain controversial.

Several randomized controlled trials (RCTs) and meta-analyses have been conducted to assess the effect of vitamin D on depressive symptoms. Although some meta-analyses have indicated that vitamin D supplementation may reduce depressive symptoms (12, 13), others have reported conflicting results (14, 15). These inconsistencies could be attributed to several factors. First, there were variations in the outcomes assessed and participant selection. Some studies have focused on depressive symptoms in healthy populations (16–18), whereas others have examined the prognosis of patients diagnosed with depression (19–21). Additionally, in certain trials, specifically individuals with vitamin D deficiency were recruited (20, 22), whereas in others, researchers did not consider this factor (19, 23). Finally, a significant heterogeneity was noted among RCTs in terms of vitamin D dosage, frequency, and route of administration (22, 24). Beyond these inconsistent results, the relationship between the dose–response effects of vitamin D supplementation remains unexplored and warrants further investigation.

In this study, we aimed to consolidate all data from RCTs involving patients diagnosed with depression who received vitamin D supplementation as an intervention. By conducting a meta-analysis, we aimed to provide robust, quantitative evidence regarding the efficacy of vitamin D supplementation in mitigating depressive symptoms in this population. Additionally, we sought to explore the dose–response relationship between vitamin D supplementation and its therapeutic effects on depressive symptoms.

## Materials and methods

### Search strategy

This study was conducted in accordance with the Preferred Reporting Items for Systematic Reviews and Meta-Analyses (PRISMA) 2020 guidelines. We conducted a systematic search of the PubMed, Embase, and Cochrane Library databases from their inception to June 2024. To maximize the search for relevant articles, we reviewed the reference lists of relevant meta-analyses published in the last 5 years. Search-term combinations used in the search strategy included ‘depression’, ‘vitamin D’, ‘randomized controlled trial’. Keywords and their synonyms were used to enhance the sensitivity of the search.

### Eligibility criteria

Studies were included in this meta-analysis if they met the following inclusion criteria: [1] RCT design; [2] trials including adult participants aged over 18 years; [3] participants diagnosed with depressive disorders according to internationally recognized diagnostic criteria (e.g., DSM-IV, DSM-5, or ICD-10), or presenting clinically significant depressive symptoms assessed using validated and widely accepted rating scales (e.g., HAM-D/HDRS, BDI/BDI-II). Depressive subtypes, including seasonal affective disorder and postpartum depression, were eligible when diagnosis or symptom severity was established using appropriate validated instruments (25); [4] trials in which the intervention was vitamin D supplementation at any dosage in oral, parenteral, intravenous, or intramuscular form, and the control group received placebo or no treatment; [5] trials in which the outcome of interest was the quantitative change in depressive symptoms assessed using depression assessment scales or questionnaires.

The exclusion criteria were as follows: (1) trials including participants aged <18 years; (2) trials enrolling pregnant women; (3) trials in which the intervention group received vitamin D in combination with additional treatments (e.g., antidepressants, exercise) that were not administered equally to the control group. Studies were included only when any co-interventions were provided identically to both groups, ensuring that the observed effects could be attributed specifically to vitamin D supplementation; (4) trials in which depression was secondary to another primary medical or psychiatric disorder (e.g., bipolar disorder), where the intervention primarily targeted the alternative condition; (5) trials that did not assess depressive symptoms using validated and standardized rating scales, or were published in non-English languages; and (6) non-randomized study designs, including cohort studies, case–control studies, case reports, conference abstracts, systematic reviews, meta-analyses, and animal studies.

### Data extraction

Articles collected through the search strategy were organized using EndNote (Version 20; Clarivate, Philadelphia, PA, United States). The retrieved articles were then screened, and data were extracted based on a pre-designed data extraction table. The extracted data from each trial included the following details: name of the first author, year of publication, country, participant characteristics (sample size, average age, serum 25-hydroxyvitamin D [25(OH)D] level at baseline and study completion), dose and frequency of vitamin D supplementation, and the duration of intervention; When multiple depression assessment scales were used in the same study, priority was given to more well-known and commonly used scales (26). Two investigators (HHL and CYL) independently collected the data for each study.

### Assessment of risk of bias

Two investigators (HHL and CYL) independently evaluated the potential risk of bias in RCTs using the Cochrane risk of bias 2.0 (27). This tool encompasses five bias domains, including the bias arising from the randomization process, bias due to deviations from the intended interventions, bias due to missing outcome data, bias in the measurement of the outcome, and bias in selecting the reported result. Each domain is rated as either having a high risk of bias, some concerns, or low risk of bias. The overall risk of bias was determined by the highest level of bias observed in any of the domains or some concerns identified in multiple domains.

### Statistical analysis

The primary outcome measure was the change in depression severity from baseline. The secondary outcomes included the change in weight; body mass index (BMI); interleukin-6 (IL-6), calcium, parathyroid hormone (PTH), high-sensitivity C-reactive protein (hs-CRP), tumor necrosis factor alpha (TNFα) levels; and clinical global impression-severity (CGI-S) scores. Although BMI and weight are not direct measures of depressive symptomatology, they were included to explore potential metabolic correlates of treatment response. The mean and standard deviation (SD) of the changes in the vitamin D and placebo groups were compared using standardized mean differences (SMDs). Random-effects meta-analyses were performed using the inverse variance method to pool the data. Heterogeneity was assessed using Cochran’s Q test and quantified using the $I^{2}$ statistic. Heterogeneity was categorized as low ( $I^{2}$ ≤ 25%), moderate (25% < $I^{2}$ < 75%), or high ( $I^{2}$ ≥ 75%) (28). All *p*-values were two-tailed, with the significance level set at 0.05. For studies reporting quartile data, mean and SD were calculated using a formula that used the values of the first quartile, median, third quartile and sample size for estimation (29, 30).

Potential variables leading to heterogeneity were investigated through subgroup analyses. These variables included the duration of intervention, participants’ baseline serum 25(OH)D levels, sex, BMI, route of administration of vitamin D supplementation, and different depression assessment scale.

All statistical analyses were performed using Review Manager software (version 5.3; Nordic Cochrane Center, Cochrane Collaboration).

In addition, we conducted a one-stage, random-effects, dose–response meta-analysis using Stata statistical software (version 18.0; College Station, TX: StataCorp LLC). The vitamin D dose was defined based on the reported daily intake in each study (e.g., 0 IU/day for the control group and 1,200 IU/day for the intervention group). Two studies were excluded as they administered extremely high doses at once (22, 31). Using Orsini’s methodology, we modeled the dose–response relationship with restricted cubic splines at the 10th, 50th, and 90th percentiles of training (32). The model used a generalized least-squares estimator to determine the correlation between effect sizes (SMD) in each study.

## Results

### Study selection

**Figure 1:**
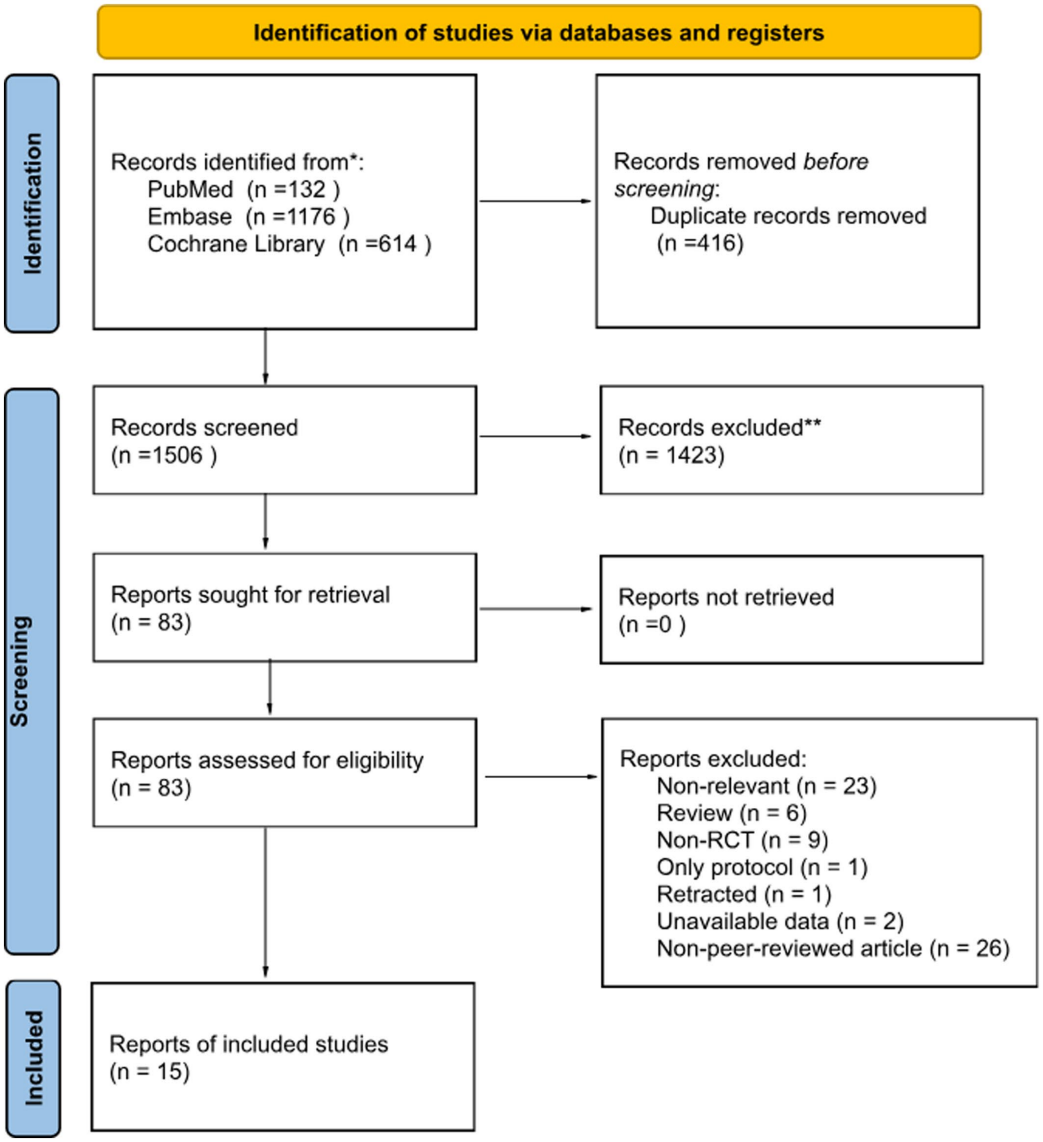
Meta-analysis flow-chart diagram according to the PRISMA guideline.

A total of 1922 relevant studies were reviewed after the database search. After excluding 416 duplicate articles, 1,506 articles were screened, and 1,423 of them were excluded based on the title and abstract. The 83 remaining articles underwent a full-text review and evaluation to assess their eligibility. Finally, 15 RCTs met the inclusion criteria and were included (22–24, 31, 33–43). The algorithm used for study selection is shown in Figure 1.

### Study characteristics

The main characteristics of the selected studies are summarized in Table 1. Participants in these studies ranged in age from 24 years to 46 years, and 501 and 461 cases were included in the experimental and control groups, respectively. The vitamin D dosage ranged from 1,000 IU per day to 12,000 IU per day. In two studies, a single high dose of vitamin D (150 000 or 300,000 IU) (22, 31) was administered. Vitamin D supplements were administered orally in most studies, except in two in which a single high-dose intramuscular injection was administered (22, 31). The duration of intervention ranged from 8 weeks to 10 months. In four studies, the intervention and control groups received treatments in addition to vitamin D, including antidepressants (33, 36, 43), cognitive behavioral therapy (36), and physical activity (35).

**Table 1:** Summary of the baseline characteristics of the included studies.

Author, years	Country	Baseline characteristic (experiment/control)				Intervention duration	Clinical symptom measurements
		Sample size	Average Age (year)	25(OH)D level (nmol/L)	Vitamin D dose, frequency, route		
Kumar et al. (43)	India.	31/28	34.9/39.3	29/28.5	60,000 IU, every 5 days, oral	12-week	HDRS-17
Kaviani et al. (42)	Iran.	28/28	43.14/42.86	87.1/73.64	50,000 IU, every 2 weeks, oral	8-week	BDI-II
Amini et al. (41)	Iran	26/24	29.25/28.92	39.83/36.74	50,000 IU, every 2 weeks, oral	8-week	EPDS
Abiri et al. (40)	Iran	26/25	34.15/34.36	41.1/40.8	50,000 IU, every week, oral	8-week	BDI-II
Zhu et al. (39)	China	62/44	46.3/43.3	39.1/42.1	1,600 IU, daily, oral	6-months	HDRS-17
Yosaee et al. (38)	Iran	27/22	38.28/37.31	65.1/51.2	2000 IU, daily, oral	12-week	BDI-II
Vellekkatt et al. (22)	India.	23/23	36.2/35.8	NR	300,000 IU, single, IM	12-week	HDRS-17
Hansen et al. (37)	Denmark	26/19	39.6/38.7	43.2/44.3	2,800 IU, daily, oral	6-months	HDRS-17
Alavi et al. (23)	Iran	39/39	68.7/67	56.3/52.9	50,000 IU, every week, oral	8-week	GDS-15
Rouhi et al. (24)	Iran	40/40	24.7/24.8	NR	1,000 IU daily, oral	10 months	EPDS
Far et al. (36)	Iran	19/20	NR	14.7	50,000 IU every week, oral	8-week	BDI-II
Far et al. (36)		13/13					
Irandoust and Taheri (35)	Iran	15/15	43.2	NR	2000 IU daily, oral	12-week	BDI
Irandoust and Taheri (35)		15/15					
Frandsen et al. (34)	Denmark	16/18	44.2/44.4	68.3	2,800 IU daily, oral	3-month	SIGH-SAD
Mozaffari-Khosravi et al. (31)	Iran.	39/34	32.1/33	NR	300,000 IU, single, IM	3-month	BDI-II
Mozaffari-Khosravi et al. (31)		36/34	32.7/33		150,000 IU, single, IM		
Khoraminya et al. (33)	Iran	20/20	38.1/39.65	58.8/57.5	1,500 IU, daily, oral	8-week	HDRS-17, BDI

NR, Not Reported; HDRS-17, Hamilton Depression Rating Scale; BDI, Beck Depression Inventory; EPDS, Edinburgh Postnatal Depression Scale; GDS-15, Geriatric Depression Scale; SIGH-SAD, Seasonal Pattern Assessment Questionnaire; IM, intramuscular injection.

### Risk-of-bias assessment

The risk-of-bias assessment of the included RCTs is shown in Supplementary Figure 1. Of 15 studies, six demonstrated an overall high risk of bias, eight demonstrated an overall moderate risk of bias, and one demonstrated an overall low risk of bias. Owing to the limitations of the study design, the lack of adherence assessment was the most common source of bias.

### Primary outcome

**Figure 2:**
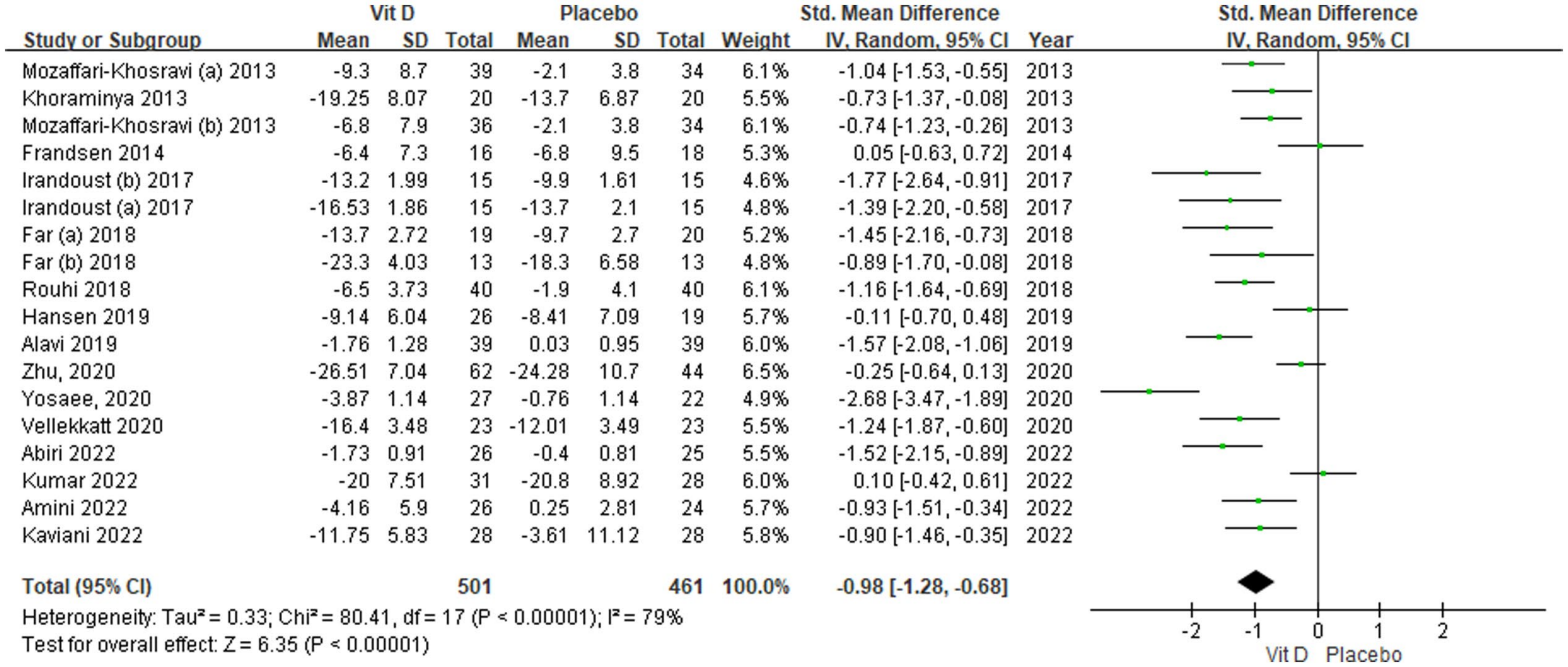
The forest plot depicts the efficacy of vitamin D supplementation in improving depressive symptoms.

An analysis of 15 RCTs involving 962 participants demonstrated that compared to placebo, vitamin D supplementation can significantly improve the symptoms of depression (SMD: −0.98; 95% CI − 1.28 to −0.68; *p* < 0.001; Figure 2). Statistical heterogeneity across the included RCTs was high (*I^2^* = 79%; *p* < 0.001).

### Secondary outcomes

The secondary outcomes included eight indicators (Table 2). Serum PTH and TNFα levels showed a significant decrease in the intervention groups than in the control groups (PTH, MD: −4.19; 95% CI − 8.18 to −0.2; pg./mL; TNFα, MD: −0.3; 95% CI − 0.44 to −0.16; pg./mL). These findings were based on data obtained from two trials for PTH (31, 42) and TNFα (40, 41). The other indicators lacked statistical significance, including weight change (MD: −0.64; 95% CI − 1.4 to 0.13; kg), BMI (MD: −0.14; 95% CI − 0.99 to 0.72; kg/ $m^{2}$ ), IL-6 (MD: 0.23; 95% CI − 0.66 to 1.12; pg./mL), serum calcium (MD: −0.17; 95% CI − 0.44 to 0.11; mmol/L), hs-CRP (MD: 0.37; 95% CI − 1.13 to 1.86; mg/L) and CGI-S scores (MD: −1.1; 95% CI − 2.71 to 0.51).

**Table 2:** Summary of secondary outcomes comparing.

Outcomes	Trials	Sample size	MD	95%Cl	$I^{2}$ (%)	*p*
Weight	5	250	−0.64	−1.4 to 0.13	26	0.24
BMI	3	165	−0.14	−0.99 to 0.72	0	0.47
IL-6	3	157	0.23	−0.66 to 1.12	53	0.12
Calcium	2	193	−0.17	−0.44 to 0.11	92	**<0.01**
PTH	2	219	−4.19	−8.18 to −0.20	0	0.67
hs-CRP	2	107	0.37	−1.13 to 1.86	63	0.1
TNFα	2	101	−0.3	−0.44 to −0.16	0	0.72
CGI-S	2	105	−1.1	−2.71 to 0.51	94	**<0.01**

BMI, body mass index; IL-6, Interleukin-6; PTH, parathyroid hormone; hs-CRP, high-sensitivity C-reactive protein; TNFα, tumor necrosis factor alpha; CGI-S, clinical global impression-severity. Bold values indicate statistical significance at *p* <.05.

### Subgroup analyses

Owing to a high heterogeneity of the results, we conducted further subgroup analyses. For the subgroup analysis of the duration of intervention, the included RCTs were divided into two groups—those with a duration longer than 8 weeks and those with a duration less than 8 weeks (Supplementary Figure 1). No significant difference was observed in depressive symptoms between the two subgroups (*p* = 0.31). Similar results were observed in the subgroup analyses of the route of administration of vitamin D supplementation (*p* = 0.95) and participants’ baseline serum 25(OH)D levels (*p* = 0.54). For the subgroup analysis of depression assessment scale, the studies were divided into two groups—those using self-rating scales such as the BDI-II and those using clinical rating scales such as the Hamilton Depression Rating Scale-17. In both groups, vitamin D supplementation effectively improved depressive symptoms. However, the extent of improvement was significantly different, with the self-rating group showing a significantly greater improvement than the clinical rating group (*p* = 0.01). In the subgroup analysis in female patients, compared with placebo, vitamin D supplementation was associated with a significantly greater improvement in depressive symptoms (SMD: −1.26; 95% CI − 1.5 to −1.01; *p* < 0.001). A similar result was observed in the subgroup analysis in patients with obesity (SMD: −1.83; 95% CI − 2.4 to −1.26; *p* < 0.001).

### Dose–response analysis

**Figure 3:**
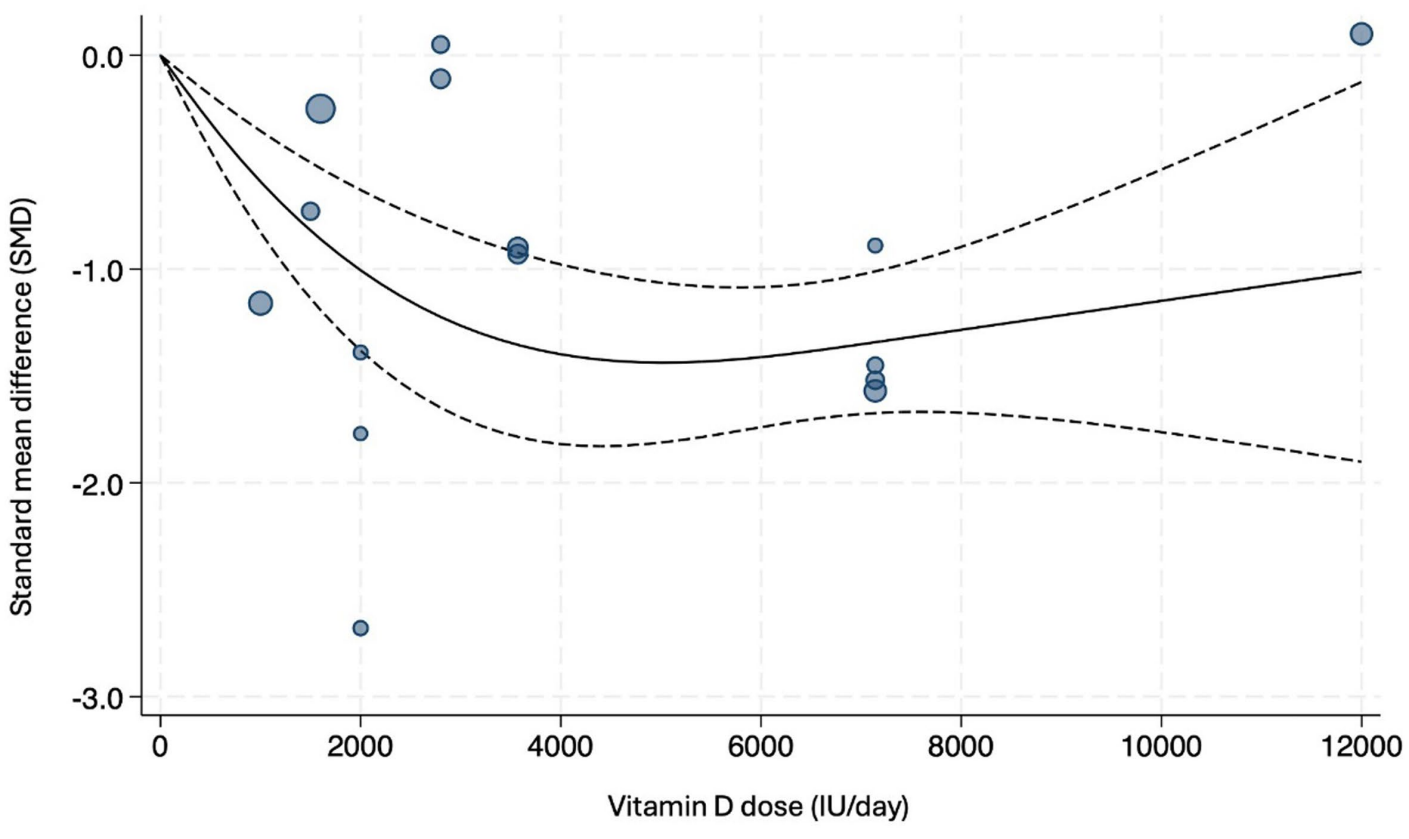
Dose–response relationship between daily vitamin D doses and SMD in depression severity.

The results of the dose–response meta-analysis are presented in Figure 3. The curve indicated a decrease in depression severity as the dosage increased. Upon reaching a daily dose of 5,000 IU of vitamin D, the SMD value reached its lowest point (SMD = −1.44, 95% CI = −1.81 to −1.06).

### Publication bias

To evaluate publication bias in our study, a funnel plot was generated (Supplementary Figure 2), which appeared symmetrical upon visual observation, indicating that no significant publication bias was observed among the included RCTs.

### Sensitivity analysis

To assess the robustness of our findings against the risk of bias, we conducted a sensitivity analysis by excluding six trials rated as having a high risk of bias (Supplementary Table 1). The analysis retained nine trials with a low or moderate risk of bias. The pooled SMD remained statistically significant and consistent with the primary outcome (SMD: −1.03; 95% CI: −1.55 to −0.51; *p* < 0.001; Supplementary Figure 3).

## Discussion

This is the first meta-analysis employing dose–response analysis to evaluate the effect of vitamin D on depression. Our study revealed several significant findings. First, the overall results of this study indicated that vitamin D supplementation significantly improves depressive symptoms in patients diagnosed with depression. Second, secondary outcomes showed that vitamin D supplementation also reduces serum PTH and TNFα levels. Third, the subgroup analyses revealed that vitamin D supplementation alleviates depressive symptoms in female patients. Similar findings were observed for patients with obesity. No significant differences were observed in the subgroup analyses of the duration of intervention, frequency, route of administration, or baseline serum 25(OH)D levels. Furthermore, the dose–response meta-analysis showed that a daily dose of 5,000 IU of vitamin D was associated with the greatest reduction in depressive symptoms within the analyzed dose range.

Previous meta-analyses examining vitamin D and depression have reported inconsistent findings (12, 14, 15, 44). These discrepancies may partly reflect differences in study populations and inclusion criteria. Several prior analyses included healthy individuals or participants with comorbid medical conditions (45, 46), whereas our study focused exclusively on patients diagnosed with depression and restricted interventions to vitamin D alone (47). These stricter criteria may provide a clearer estimate of the adjunctive effect of vitamin D in this population.

The secondary outcomes demonstrated reductions in serum PTH and TNFα levels following vitamin D supplementation. However, these findings were derived from only two trials per biomarker and should therefore be interpreted cautiously. While the decrease in TNFα may suggest a potential role of inflammatory modulation in the antidepressant effects of vitamin D, the limited evidence base renders these results preliminary and hypothesis-generating rather than confirmatory. Although inflammation has been implicated in the pathophysiology of depression (48, 49), our meta-analysis was not designed to directly evaluate mechanistic pathways, and causal inferences cannot be drawn.

The subgroup analyses revealed that patients with obesity could benefit from vitamin D supplementation for alleviating depressive symptoms. A previous study showed that the bioavailability of vitamin D in individuals with obesity is lower than that in healthy individuals (50), which may be related to the deposition of vitamin D in body fat. Conversely, vitamin D supplementation may indirectly alleviate depressive symptoms associated with obesity by improving insulin sensitivity and metabolic function (51), which may explain why vitamin D supplementation remained effective for patients with obesity in our study. It is also important to acknowledge that obesity is associated with altered vitamin D pharmacokinetics and a pro-inflammatory metabolic milieu, which may influence treatment responsiveness (52). Although randomization within individual trials minimizes internal confounding, these biological factors may contribute to between-study heterogeneity and limit generalizability beyond this population.

Our study also showed that vitamin D supplementation could improve depressive symptoms in female patients. Several epidemiological studies have shown that the risk of depression in women is approximately twice that in men, potentially due to physiological mechanisms such as hormonal fluctuations and differences in brain neurotransmitter systems (53). The serotonin system in women is particularly susceptible to hormonal fluctuations, and vitamin D may enhance serotonin synthesis and availability in the brain (54), thereby improving depressive symptoms. Another potential mechanism involves estrogen regulation. Some women are sensitive to normal fluctuations in estrogen levels, which may be associated with female-specific reproductive types of depression (53), including post-partum and post-menopausal depression. Vitamin D stabilizes estrogen levels by regulating estrogen’s interaction with its receptors (55) or enhancing estrogen synthesis (56), thus contributing to the alleviation of depressive symptoms.

Additionally, the subgroup analyses showed that assessment using different depression assessment scales affected outcomes. The groups using self-rating scales exhibited greater improvements in depressive symptoms than the group using clinical rating scales. This suggests the presence of unrecognized biases in the blinding methods of some of the included studies. Another possible explanation is related to self-efficacy associated with self-rating scales. Compared with clinical rating scales, patients using self-rating scales tend to pay more attention to their emotional changes, allowing them to reflect on their improvement during a treatment. This process can enhance patients’ engagement in the treatment plan, making them more sensitive and optimistic about treatment outcomes, and ultimately leading to higher self-rating scores (57, 58). In addition, clinical rating scales tend to focus more on objective physical symptoms, such as weight loss or changes in appetite (58). The therapeutic effects of vitamin D supplementation may not be immediately reflected in these symptoms, which may result in underestimation of the effectiveness of vitamin D.

The result of dose–response meta-analysis indicated that a daily intake of 5,000 IU of vitamin D may yield optimal therapeutic effects against depression. Recent clinical research suggests that individuals with depression, particularly those with vitamin D deficiency, may benefit from higher doses (59, 60). Depression is frequently linked to altered vitamin D metabolism and is influenced by factors such as inflammation, metabolic comorbidities, and baseline vitamin D levels (51, 61). These physiological considerations in patients with depression may justify a customized supplementation approach beyond standard guidelines. Although a daily intake of 5,000 IU exceeds the commonly cited tolerable upper intake level for the general adult population, safety evaluations in clinical contexts have suggested that higher intakes may be acceptable under appropriate monitoring. Nevertheless, the applicability of this dosage may vary depending on baseline vitamin D status, comorbid conditions, and individual metabolic differences (62, 63). Importantly, safety concerns remain for certain subpopulations. Individuals with granuloma-forming disorders or specific lymphomas may exhibit heightened sensitivity to vitamin D due to unregulated extrarenal production of 1,25-dihydroxyvitamin D, which can precipitate hypercalcemia even at relatively lower doses (64). Therefore, extrapolation of this dosage to individuals without confirmed vitamin D deficiency or outside supervised clinical settings should be approached with caution. High-dose supplementation strategies should be individualized and accompanied by regular monitoring of serum 25(OH)D and calcium concentrations.

This study has several limitations. First, the meta-analysis demonstrated substantial heterogeneity in the primary outcome, which warrants cautious interpretation of the pooled effect size. This heterogeneity likely stems from variations in study design, vitamin D dosage and administration frequency, diversity within the study populations, and differences in depression assessment scales. However, our subgroup analyses explored potential contributors to the observed variability. Heterogeneity appeared reduced in certain subgroups, including female participants, shorter-duration interventions, and trials employing intramuscular administration. While these findings suggest greater consistency of effect within specific contexts, they should be interpreted cautiously given the limited number of studies and the exploratory nature of subgroup analyses. Second, although some included trials involved adjunctive treatments such as antidepressants, psychotherapy, or physical activity, these interventions were administered equally in both intervention and control groups in accordance with our inclusion criteria. Therefore, the randomized comparisons within each study preserved the internal validity of the estimated vitamin D effect. However, due to inconsistent reporting and limited variability in adjunctive treatment protocols across trials, we were unable to conduct formal subgroup analyses or meta-regression to explore potential interaction effects. Residual confounding related to background therapies cannot be entirely excluded and should be considered when interpreting the findings. Third, the methodological quality of included trials varied, with several studies assessed as having moderate or high risk of bias. Such limitations may affect confidence in the pooled estimates and raise the possibility of effect size inflation. However, sensitivity analyses excluding high-risk studies yielded results that were consistent in direction and magnitude with the primary analysis. While this supports the stability of the overall findings, the presence of methodological limitations warrants cautious interpretation and underscores the need for further well-designed and adequately powered randomized controlled trials. Fourth, the inclusion of diverse depressive subtypes, such as postpartum depression and seasonal affective disorder. Although all participants met recognized diagnostic criteria or had clinically significant depressive symptoms, these conditions differ in underlying mechanisms and treatment responsiveness. Pooling them may have introduced clinical heterogeneity. Therefore, our findings should be interpreted as reflecting an overall effect across depressive disorders rather than subtype-specific efficacy. Future trials and meta-analyses focusing on more homogeneous diagnostic categories would help clarify whether treatment effects differ across depressive subtypes. Finally, external factors, such as dietary habits, sun exposure, and baseline serum 25(OH)D levels, are known to influence the efficacy of vitamin D supplementation. Although these factors are critical for understanding the broader effects of supplementation, not all studies included in our analysis provided comprehensive data on these variables, which may have contributed to the variability in our results.

In conclusion, vitamin D supplementation may serve as a promising adjunctive strategy for reducing depressive symptoms in patients with depression. Greater improvements were observed in specific subgroups, and reductions in TNFα suggest a potential anti-inflammatory role. Higher daily intakes, particularly around 5,000 IU, were associated with greater symptom improvement; however, further high-quality trials are required to confirm optimal dosing and long-term safety.

## Data Availability

The original contributions presented in the study are included in the article/Supplementary material, further inquiries can be directed to the corresponding authors.
