# Supplementary material for: Efficacy of vitamin D supplementation in patients diagnosed with depression: a dose–response meta-analysis of randomized controlled trials
Source: Front Nutr. 2026 Mar 17;13:1772451. doi: 10.3389/fnut.2026.1772451 (PMC13035762; doi:10.3389/fnut.2026.1772451)

Supplementary

Supplementary Table 1. Risk-of-bias assessment of the included RCTs

| Study | D1 | D2 | D3 | D4 | D5 | Overall |
| --- | --- | --- | --- | --- | --- | --- |
| Kumar, 2022 | Some concerns | Some concerns | Low | Low | Some concerns | Some concerns |
| Kaviani, 2022 | Some concerns | Low | Some concerns | Low | Low | Some concerns |
| Amini, 2022 | Low | Low | Low | Low | Some concerns | Some concerns |
| Abiri, 2022 | Low | Low | Low | Low | Low | Low |
| Zhu, 2020 | High | High | Some concerns | Some concerns | Some concerns | High |
| Yosaee, 2020 | Some concerns | Low | Some concerns | Low | Some concerns | Some concerns |
| Vellekkatt, 2020 | Low | Low | Low | Low | Some concerns | Some concerns |
| Hansen, 2019 | Low | High | Some concerns | Low | Low | High |
| Alavi, 2019 | Low | Low | Low | Low | Some concerns | Some concerns |
| Rouhl, 2018 | Low | High | Low | Low | Some concerns | High |
| Far, 2018 | High | High | Low | High | Some concerns | High |
| Irandoust, 2017 | High | High | Low | High | Some concerns | High |
| Frandsen, 2014 | Low | Low | Some concerns | Low | Some concerns | Some concerns |
| Mozaffari-Khosravi, 2013 | High | High | Some concerns | Some concerns | Some concerns | High |
| Khoraminya, 2012 | Low | Low | Low | Low | Some concerns | Some concerns |

Supplemental Figure 1. The forest plot shows subgroup analyses of the depressive symptoms according to duration of intervention, frequency and route of administration of vitamin D supplementation, participant’s level serum 25(OH)D, different depression assessment scale, BMI, sex.


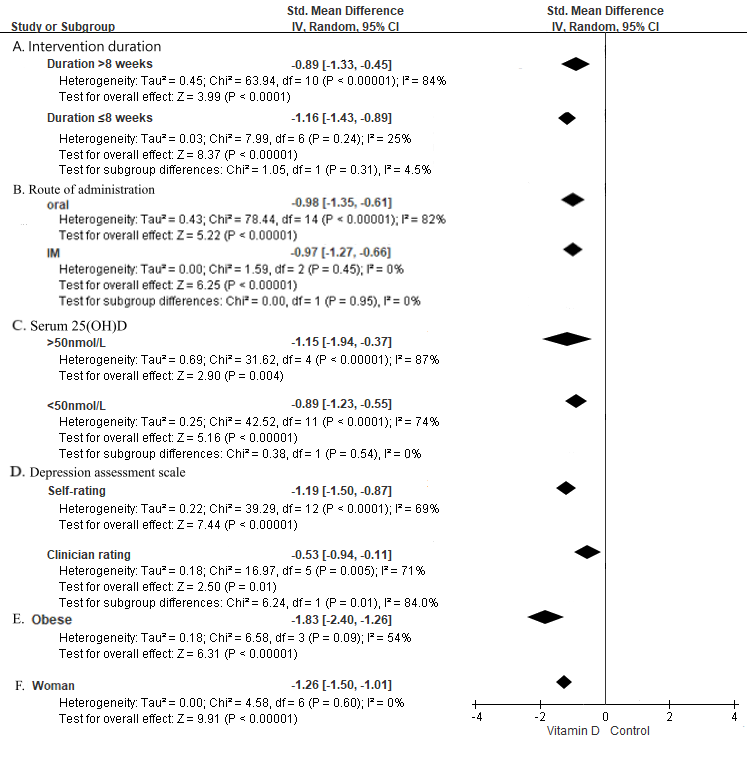


Supplementary Figure 2. Funnel plot


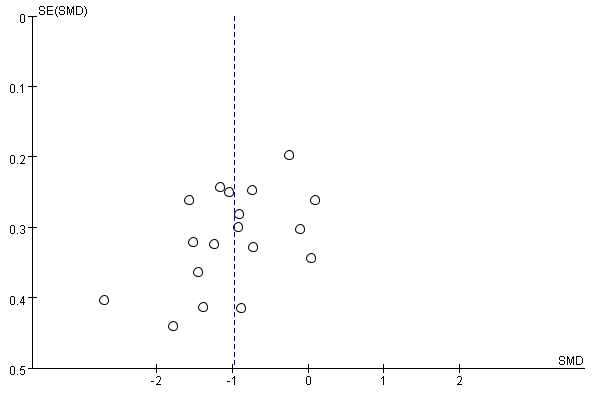


Supplementary Figure 3. Sensitivity analysis excluding six studies rated as having high risk of bias


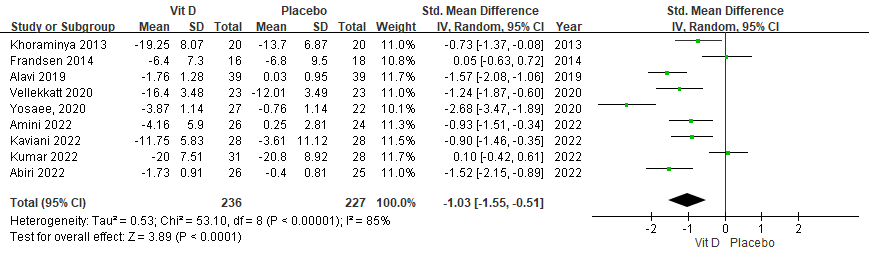

Supplement: Supplementary file 1 [file Table_1.docx]
